# Revised Aspects into the Molecular Bases of Hydroxycinnamic Acid Metabolism in Lactobacilli

**DOI:** 10.3390/antiox12061294

**Published:** 2023-06-17

**Authors:** Félix López de Felipe

**Affiliations:** Laboratorio de Biotecnología Bacteriana, Instituto de Ciencia y Tecnología de los Alimentos y Nutrición (ICTAN), CSIC, José Antonio Novais 10, 28040 Madrid, Spain; fxlopez@ictan.csic.es

**Keywords:** hydroxycinnamic acids, metabolism, molecular bases, *Lactobacillus*

## Abstract

Hydroxycinnamic acids (HCAs) are phenolic compounds produced by the secondary metabolism of edible plants and are the most abundant phenolic acids in our diet. The antimicrobial capacity of HCAs is an important function attributed to these phenolic acids in the defense of plants against microbiological threats, and bacteria have developed diverse mechanisms to counter the antimicrobial stress imposed by these compounds, including their metabolism into different microbial derivatives. The metabolism of HCAs has been intensively studied in *Lactobacillus* spp., as the metabolic transformation of HCAs by these bacteria contributes to the biological activity of these acids in plant and human habitats or to improve the nutritional quality of fermented foods. The main mechanisms known to date used by *Lactobacillus* spp. to metabolize HCAs are enzymatic decarboxylation and/or reduction. Here, recent advances in the knowledge regarding the enzymes that contribute to these two enzymatic conversions, the genes involved, their regulation and the physiological significance to lactobacilli are reviewed and critically discussed.

## 1. Introduction

The hydroxycinnamic acids (HCAs) are a family of phenolic compounds of vegetal origin that are present in a wide variety of edible plants [1]. HCAs have been largely connected to potential health benefits, as they can act as powerful antioxidants and thus protect against diseases associated with oxidative stress and genotoxicity, mainly by scavenging a variety of reactive oxygen species (ROS), though they can also inhibit enzymes promoting oxidative stress (such as ROS/RNS-generating enzyme) [2]. The antioxidant or radical scavenging capacity of HCAs is inversely correlated with their redox potential, so that the HCAs displaying higher antioxidant activity have lower redox potentials [3] (Table 1).

The genus *Lactobacillus* encompasses well-studied and very diverse species. Recently, a polyphasic approach has permitted the reclassification of the genus *Lactobacillus* into 25 genera, including an emended description of the genus *Lactobacillus* and 23 novel genera. This reclassification allows lactobacilli to be grouped into clades with shared ecological and metabolic properties [4].

### Antimicrobial Stress on Lactobacilli Associated with Hydroxycinnamic Acids

In addition to their antioxidant properties, a known biological effect of HCAs involves strong antimicrobial activity against Gram-positive and Gram-negative bacteria [5,6]. The HCAs, as weak acids, can passively enter into the cell and acidify the cytoplasm, but lactobacilli, in general, are highly protected against acid stress. This is, in part, due to an F-ATPase that displays optimum activities at low pH values [7]. However, variations are not observed in the levels of F-ATPase in the responsive transcriptome of *Lactiplantibacillus plantarum* [8] and *Levilactobacillus brevis* [9] or proteome of *Lacticaseibacillus casei* [10] to different HCAs, indicating that acid stress is not a principal mechanism underlying the antimicrobial action of HCAs on lactobacilli.

Since antimicrobials usually share the production of ROS to kill bacteria [11], it is fair to propose that one of the mechanisms underlying the antimicrobial activity of HCAs can be based on their capacity to act as pro-oxidant agents. As hydroxylated aromatics and electrochemically active compounds, HCAs can be oxidized to their corresponding semiquinones and quinones in the presence of appropriate electron acceptors (mainly ubiquitous oxygen) with the formation of superoxide and hydrogen peroxide. The pro-oxidant behavior of HCAs can be complex and is dependent on many factors, including concentration, pH, type of substrate or presence of metal chelating properties [12]. Regarding the concentration factor, it is important to note that antioxidant effects of HCAs have been found in µM concentrations [12]. However, it has been proposed that above concentrations of 5 µM, these compounds begin to promote a pro-oxidant behavior [12], and higher concentrations (up to mM) are used in experiments aimed to test the antimicrobial capacities of HCAs [6]. The oxidation rate of HCAs is accelerated in the presence of ubiquitous redox-active metals (Mn, Fe, Cu), which can form complexes with the HCAs and serve as catalysts to facilitate the electron transfer to dioxygen, thus generating organic radicals along with H_2_O_2_, such as during the oxidation of caffeic acid by Cu^2+^ [12]. Hydrogen peroxide is converted by the Fenton reaction to destructive superoxide anions and hydroxyl radicals that damage the membrane and biological macromolecules. In addition, it is important to note that the semiquinone and quinone formed as products during the oxidation of HCAs can be powerful oxidants and can act as catalysts to accelerate autoxidation [13]. The semiquinone radicals and the superoxide formed when the HCAs react with dioxygen are also able to damage biological membranes.

The damage caused in the cell envelope of lactobacilli by ROS arising from the pro-oxidant activity of HCAs adds to their amphipathic nature, which enables these compounds to insert into and destabilize the membrane. This damage is reflected in the studied transcriptomic responses of *L. plantarum* WCFS1 [8] or the proteomic responses of *L. casei* BL23 [10] exposed to *p*-coumaric acid (*p*-CA), or in the ferulic acid-responsive transcriptome of *L. brevis* [9]. These global responses include, besides other adaptations, a variable regulation of genes and proteins involved in membrane lipid, phospholipid and cell-wall biosynthesis to counter this stress [14]. The pro-oxidant activity associated with *p*-CA is revealed by the remarkable transcriptional response elicited by *L. plantarum* WCFS1 to cope with oxidative stress in the presence of this compound [8]. However, the superoxide dismutase (SOD), a crucial enzyme to protect cells against oxidative stress, is not present in the genome of *L. plantarum* and is not widely distributed among lactobacilli. SOD can remove the superoxide (O_2_**^−^**) formed during the (auto)oxidation of HCAs, hence its absence in many lactobacilli could increase the oxidative burden arising from the presence of HCAs inside lactobacilli. Due to the lack of SOD, many lactobacilli require high levels of manganese for fermentation and aerobic growth [15]. However, Mn^3+^, which is generated by O_2_^−^ from Mn^2+^, can dramatically increase the rate of autooxidation of quinones and the concomitant production of H_2_O_2_ [16]. Since the generation of Mn^3+^ is inhibited by SOD, the absence of this enzyme may further increase the oxidative burden imposed by the oxidation of HCAs, as superoxide radicals cannot be efficiently removed and the quinone forms of HCAs cannot be maintained in their reduced state. In *L. plantarum*, the response to the oxidative stress induced by *p*-CA involves the induction of the thioredoxin reductase system, the glutathione reductase system, the methione sulfoxide reductase and the expression of a broad set of genes dedicated to methionine biosynthesis [8]. Although this thiol-specific oxidative stress response dedicated to repair proteins and repair membrane lipids can attenuate the damages induced by the ROS generated during the oxidation of HCAs, it is possible that such damages cannot be fully prevented due to the low prevalence of SOD among *Lactobacillus* spp. and the absence of induction of the NADH oxidase/NADH peroxidase system by these phenolic compounds.

As tools to tackle the stress imposed by these compounds, some lactobacilli are able to metabolize the HCAs by enzymatic decarboxylation and/or reduction. Here, knowledge on the enzymes and genes involved in these metabolic conversions, their regulation and the physiological significance to lactobacilli is reviewed and critically discussed.

## 2. Decarboxylation of Hydroxycinnamic Acids

Some *Lactobacillus* spp. are known to decarboxylate hydroxycinnamic acids (HCAs) to their corresponding vinyl derivatives, which are considered less toxic than their precursors. This metabolic conversion is catalyzed by the phenolic acid decarboxylase (EC.4.1.1.) (PAD, also named PDC), an enzyme that can be inducible at the transcriptional level by the substrate, the HCAs. Thus, the gene encoding this enzyme, *pad*, was found to be highly induced at the transcriptional level (more than 100 times induction with respect to the control) in the presence of *p*-CA in several *L. plantarum* strains [8,17] as well as in the presence of ferulic acid in *L. brevis* [8]. The *pad* gene is also markedly upregulated in some *Lactiplantibacillus pentosus* strains in the presence of *p*-CA, ferulic or caffeic acids [18], or by ferulic or caffeic acids (but not sinapic acid) in *Furfurilactobacillus rossiae* [17]. However, the induction of the *pad* gene by HCAs in *L. pentosus* [18] and *F. rossiae* is strain-dependent [17]. In the case of the *Limosilactobacillus fermentum* FUA3589 strain, it has been reported that the chromosome-encoded *pad* gene is not induced in the presence of HCAs [17]. Of note, a recent search among all sequenced *Lactobacillus* spp. has shown that not all species contain *pad* in their chromosomes [17]. Examples of *pad*-lacking lactobacilli include *Limosilactobacillus reuteri*, *Lactobacillus delbrueckii* and *Ligilactobacillus salivarius* groups [17], as well as *Lacticaseibacillus casei* BL23 [10].

**Figure 1:**
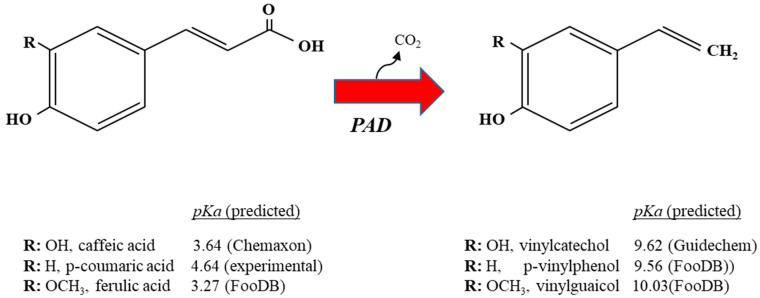
Graphic representation of metabolic decarboxylation of caffeic, *p*-coumaric and ferulic acid into their corresponding vinyl derivatives by *Lactobacillus* spp. The predicted p*K*a of the hydroxycinnamic acids and their vinyl derivatives are shown. Each vinyl derivative shares the same R group as the hydroxycinnamic acid from which it is derived; PAD, phenolic acid decarboxylase.

Despite the variability in *pad* expression observed among lactobacilli, the striking HCA-dependent induction of the *pad* gene and the failure of a *pad L. plantarum* mutant to grow in the presence of relatively high (above 3 mM) *p*-CA concentrations [19] show that decarboxylation is a key mechanism to relieve the toxicity of HCAs, at least in lactobacilli that hold and express the *pad* gene, such as *L. plantarum* spp. Following decarboxylation, several HCA properties can be altered that may reduce the stress associated with HCAs. One of the advantages for lactobacilli that results from the enzymatic decarboxylation of HCAs by the PAD is that the produced vinyl derivatives, typically vinyl phenol, vinyl catechol and vinyl guaycol, from *p*-CA, caffeic and ferulic acids, respectively, display lower acidity than their corresponding HCA precursors (examples of vinyl derivatives and their precursors are vinyl phenol (p*K*a, 9.56), *p*-CA (p*K*a, 4.64), vinyl catechol (p*K*a, 9.62), caffeic acid (p*K*a, 3.64), vinyl guaicol (p*K*a, 10.03) and ferulic acid (p*K*a, 3.27). (Figure 1).

In addition, the carboxylic group of HCAs has been demonstrated to act as the binding site for metal ions, at least for *p*-CA [20]. As mentioned in the Section 1, redox cycling of HCAs can be catalyzed by redox-active metals in the presence of oxygen to produce organic radicals and ROS. Since carboxylic groups provide effectiveness in complexing metals [12], the enzymatic decarboxylation provided by the PAD would reduce the binding of metal ions to HCAs, thus diminishing its potential pro-oxidant behavior.

Another property of HCAs that can be altered by the decarboxylation mediated by the PAD is their lipophilicity. The elimination of the carboxylic group (COOH) in the structure of HCAs may prevent the increase of the lipophilicity of HCAs arising from their esterification. Esterification involves the carboxylic group and increases the lipid solubility of HCAs [21]. In addition, the carboxylic group is able to form complexes with hydroperoxides, thus accelerating the kinetics of lipid oxidation [22], particularly at low pH [23] which is an environmental condition usually faced by lactobacilli.

As commented above, and despite the potential advantages that the decarboxylation reaction may confer to lactobacilli to counter the toxicity of HCAs, not all *Lactobacillus* spp. and strains possess PAD. Moreover, even if they hold the *pad* gene in their chromosomes, some of them, such as the *L. fermentum* FUA3589 strain, do not induce this gene in the presence of HCAs.

However, lactobacilli have developed other strategies to counter the toxicity of HCAs, including thiol-specific stress responses, activation of general stress pathways, overexpression of MFS-type [8,9] or ABC-type [8] multidrug extrusion systems, metabolic adaptations to enter into carbohydrate starvation conditions and membrane as well as cell-wall modifications to stabilize and reinforce the cell envelope. These mechanisms are described in more detail in a previous review [14].

It is important to note that, though HCAs are grouped as the same class of phenolic compounds, they have different physico-chemical characteristics, including different reduction potentials. For example, *p*-CA displays a standard reduction potential of 739 mV while FA and CA have 447 mV and 183 mV potentials, respectively. Therefore, *p*-CA is more prone to act as a pro-oxidant than FA, which is more prone than CA [12]. Accordingly, the pro-oxidative effects of HCAs would differ among them, and therefore different strategies should be expected to be used by lactobacilli to counteract the antimicrobial effects of these different phenolic compounds. Thus, it has been observed that the transcriptomic response of *L. plantarum* in the presence of *p*-CA [8] involves many more genes dedicated to countering oxidative stress than *L. brevis* in the presence of ferulic acid [9]. In this line, the differences in the structure of the different HCAs have been recently proposed to determine, together with the specific strain, the phenolic acid metabolism in the probiotic *Lacticaseibacillus rhamnosus* GG, *L. plantarum* 299 v and *L. reuteri* DSM 17938 strains [24].

**Figure 2:**
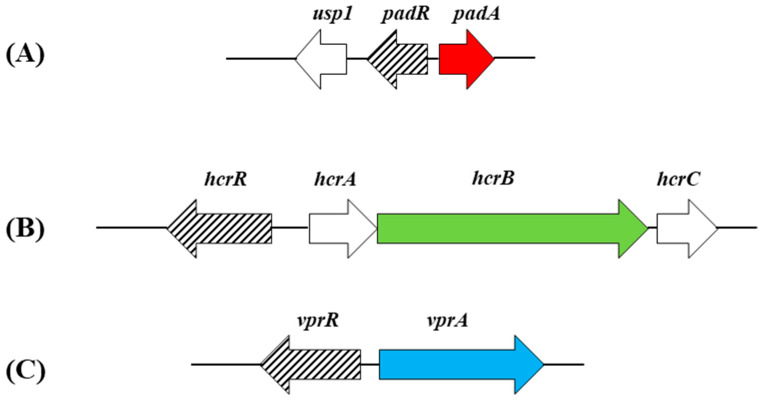
Genetic organization of the *L. plantarum* WCFS1 chromosomal regions containing clusters harboring genetically and biochemically validated genes determining hydroxycinnamate metabolic conversions: hydroxycinnamate decarboxylation *padA* or *lp_3665* (red arrow) (**A**) [NCBI accession NC_004567; *lp_3663* (or *usp1*) through *lp_3665* (or *padA*)]; hydroxycinnamate reduction *hcrB* or *lp_1425* (green arrow) (**B**) [NCBI accession NC_004567; *lp_1422* (or *hcrR*) through *lp_1426* (or *hcrC*)] or vinylphenol reduction *vprA* or *lp_3125* (blue arrow) (**C**) [NCBI accession NC_004567; *lp_3124* (or *vprR*) through *lp_3125* (or *vprA*)]. Lys-R type regulators that control decarboxylation or reduction of hydroxycinnamates as well as the reduction of vinylphenols, are indicated by lined arrows.

The production of vinyl derivatives from HCAs by enzymatic decarboxylation can be subjected to regulation in lactobacilli. In *L. plantarum*, the *pad* gene is specifically controlled by PadR, a transcriptional regulator (repressor) encoded by the contiguous and divergently oriented *padR* gene (Figure 2).

A PadR consensus dyad sequence named IR1-2 (ATGT-8N-ACAT) has been previously proposed to permit the binding of this repressor in *Bacillus subtilis* [25]. The IR1-2 dyad element has been found in the promoter regions of genes from Gram-positive and Gram-negative bacteria that are involved and not involved in the phenolic acid stress response (PASR), which suggests that PadR is a pleiotropic regulator [25]. However, it was reported that, in *B. subtilis*, the IR1-2 dyad element overlaps with a second dyad element named dIR1-2bis [25]. The entire dIR1-2bis/IR1-2 sequence permits binding of two PadR dimers, which may be observed in bacteria grown under noninduced conditions where the promoter of the phenolic acid decarboxylase gene is completely repressed. Based on the in silico identification of putative binding sites for PadR in *L. plantarum* WCFS1, it has been observed that the IR1-2 dyad element is located in the promoter regions of 40 genes, from which only three were involved in the PASR. When the search in silico for PadR-putative binding sites included a second dyad element dIR1-2bis designed for *L. plantarum*, the entire dIR1-2bis/IR1-2 sequence was uniquely identified in the region lying upstream of *padR* and *padC* sequences of the genome of *L. plantarum* WCFS1, and therefore was proposed as the interacting sequence with PadR that completely represses the *padC* promoter [8]. In the phenolic acid stress response (PASR) of *B. subtilis*, the repressor of *padC*, PadR, is inactivated by these acids [2], which suggests that this mechanism is also behind the huge overexpression of *pad* in the *Lactobacillus* spp described above. *L. brevis* also displays a *padR* gene contiguously located and divergently oriented to *pad* [9], which indicates a similar transcriptional control to *L. plantarum* WCFS1.

Several *Lactobacillus* PADs have been purified and characterized to date, and differences at the biochemical level have been reported between these enzymes. Thus, the PAD from *L. plantarum* CECT 748T strain (identical in its amino acid sequence to the type strain *L. plantarum* WCFS1) acts only on *p*-CA, caffeic and ferulic acids [26], while the PAD from *L. plantarum* LPCHL2 decarboxylates a broader range of hydroxycinamic acids [27], with these differences in substrate utilization attributed to differences in their respective C-terminal regions. In any case, both *L. plantarum* enzymes preferentially decarboxylate *p*-CA and caffeic acid according to their determined *K_m_* values for HCAs. The PAD from *L. brevis* RM84 has been also purified and characterized [28]. This enzyme decarboxylates HCAs, and it shows similar *K_m_* values for *p*-CA, caffeic and ferulic acids.

## 3. Reduction of Vinyl Derivatives of Hydroxycinnamic Acids into Ethyl Derivatives

**Figure 3:**
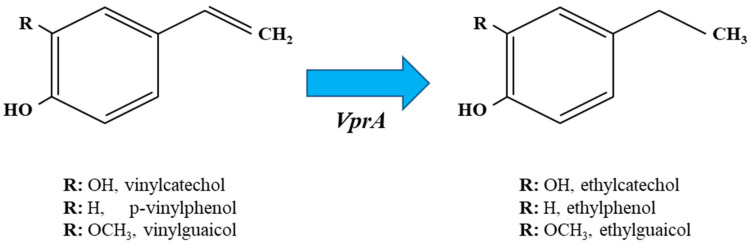
Graphic representation of metabolic reduction of vinyl derivatives of caffeic (vinylcatechol), *p*-coumaric (vinylphenol) and ferulic (vinylguaicol) acids by *Lactobacillus spp*. Each ethyl derivative shares the same R group as the vinyl derivative from which it is derived. VprA, vinylphenol reductase A.

The vinyl derivatives produced by the decarboxylation of HCAs, typically vinyl phenol, vinyl catechol and vinyl guaycol from *p*-coumaric, caffeic and ferulic acids, respectively, can be reduced by *Lactobacillus* spp. to its corresponding ethyl derivatives (4-ethyl phenol, 4-ethyl catechol and 4-ethyl guaycol, respectively) (Figure 3). The enzyme that catalyzes this reaction is the vinyl phenol reductase (EC.1.3.1.74) (VprA), which has recently been genetically and biochemically characterized but solely in *L. plantarum* WCFS1 [29]. The expression of the *vprA* gene is controlled by the Lys-R type regulator VprR (Figure 2), as demonstrated by the lack of ethylphenol production in a *vprR L. plantarum* mutant.

It must be noted that the reduction of vinyl derivatives to ethyl derivatives seems to be rather limited among lactobacilli, since *vprA* is not widely distributed among these microorganisms and other lactic acid bacteria [17,29]. It has been also observed that about half of the *Lactobacillus* strains studied that encode VprA did not bear the gene coding for PAD [17]. In addition, in spite of the fact that the *L. plantarum pad* gene shows a huge natural overexpression in response to *p*-CA, the conversion of *p*-CA to vinylphenol under anaerobic conditions was slow [30]. Accordingly, *L. plantarum*, which holds PAD and VprA, produces ethylphenol more efficiently if vinylphenol is exogenously provided than if using *p*-CA as precursor; under the anaerobic conditions being described, the reaction catalyzed by PAD is a limiting factor for the production of ethylphenol [30]. It should also be noted that the *vprA* and *pad* genes are distant in the chromosome and are under the control of different regulators, namely VprR and PadR, respectively (Figure 2). Altogether, these data show that PAD and VprA can function independently and suggest that they do not necessarily catalyze steps of the same metabolic pathway.

Although not frequent among lactobacilli, the conversion of vinyl derivatives of HCAs into ethyl derivatives is of interest in the food industry, as ethylphenols are strong odorants known to influence, even at low threshold values, the organoleptic quality of wine, beer or ciders and fermented foods such as sauerkraut where the core lactic acid bacteria involved hold the *vprA* gene [31]. The microbial production in the gut of ethylphenols has also attracted attention related to the host health, as the host-sulfated derivative of 4-ethylphenol, the 4-ethylphenyl sulphate, has been demonstrated to be a neuroactive microbial molecule that impacts brain activity and complex behaviors in animals [30]. In addition, the 4-ethylcatechol has been proposed to contribute to reducing the risk of colon cancer [32]. The precursor of ethylphenol, vinylphenol, is also known to influence behavior in plants [33], which previously raised the question of whether vinyl derivatives of HCAs can also impact behavior in animals [8]. Accordingly, knowledge of the toolkit of lactobacilli that enable these bacteria to convert HCAs into vinyl derivatives and ethyl derivatives, and the regulation of these metabolic conversions, still deserves further investigation, as it can be important to design strategies to control the production of these odorants in foods or as beneficial microbial molecules in humans and animals.

## 4. Reduction of Hydroxycinnamic Acids

**Figure 4:**
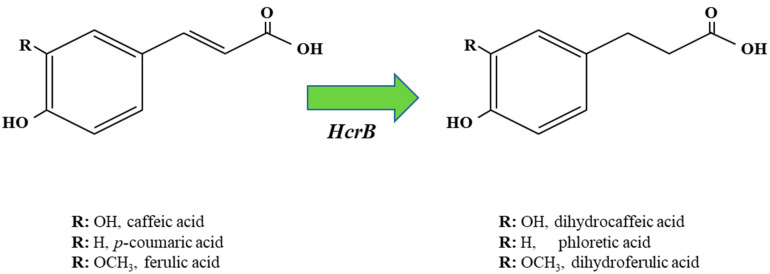
Graphic representation of metabolic reduction of caffeic, *p*-coumaric and ferulic acid into phenylpropionic derivatives by *Lactobacillus* spp. HcrB, hydroxycinnamate reductase B. Each phenylpropionic acid shares the same R group as the hydroxycinnamic acid from which it is derived.

In addition to, or alternatively to, decarboxylation some *Lactobacillus* spp. display the capacity to reduce hydroxycinnamic acids into phenylpropionic acids [17,34] (Figure 4). Among lactobacilli, the enzymes involved in this reduction process have been solely validated from a biochemical viewpoint in *L. plantarum*, and are encoded by the *hcrA* and *hcrB* genes (Figure 2). Whole-genome transcriptional profiling studies have shown that the expression levels of the *hcrAB* genes are strongly elevated in the presence of *p*-CA [8]. The *hrcB* transcripts were also overexpressed in pineapple juices [35], a plant food matrix rich in hydroxycinamic acids [36]. The *L. plantarum* hydroxycinamate reductase (HCR) proteins (HcrAB) encoded by the *hcrAB* genes have been recently purified and characterized [37]. The characterization of the HCR activity by these enzymes has been mainly performed by using *m*-coumaric acid as a model HCA, as *L. plantarum* does not metabolize this compound by decarboxylation but only by reduction to 3-(3)-hydroxyphenyl)propionic acid (3-HPPA) [34]. It was determined that HcrA did not exhibit HCR catalytic activity in vitro in the presence of the required FMN and NADH cofactors. However, this protein improved HcrB production when both enzymes were coproduced together. In addition, the coproduction of these enzymes reduced the partial proteolysis of HcrB (and its subsequent partial inactivation), observed when this protein was purified alone [37]. It was then proposed that the role of HcrA is to stabilize and facilitate the solubility of the heterodimer that this protein forms with HcrB [37], similarly to other coupled FMN reductases described in *Lactobacillus acidophilus* [38] and *Lactobacillus johnsonii* [39]. However, up to now it is unknown if the NAD(P)H-binding domain (COG0431) present in HcrA serves to bind a nicotinamide cofactor and if it participates in the electron transfer on the way to the final acceptor by the HcrAB heterodimer, as it occurs with the COG0431 domain of the contiguous HcrB, an enzyme that has been shown to require and bind NADH for activity.

By using extracts from *Escherichia coli* overexpressing the recombinant HcrB protein, instead of using the purified HcrB protein, it was possible to relieve its partial hydrolysis and inactivation and determine that HcrB (EC.1.3.1.11) constituted the minimum requirement for HCR activity [37]. Thus, the HcrB protein was demonstrated to act on the unsaturated bond of the ethylenic side chain of HCAs to reduce (although only partially) the *p*-coumaric, *m*-coumaric, *o*-coumaric, ferulic, caffeic and sinapic acids into their corresponding phenylpropionic acid derivatives [37].

In spite of the presence of the double bond in the aliphatic side chain of HCAs possibly being expected to provide greater H-donating ability and subsequent radical stabilization, some phenylpropionic acid derivatives, such as dihydrocaffeic acid, seem to confer a slightly improved resistance to oxidative stress compared to caffeic acid [40], and show a radical scavenging activity higher than caffeic acid [41]. However, this property is dependent on the type of HCA [42] and the system where the oxidation happens [40]. Therefore, it is not clear whether the reduction of HCAs into HPPA derivatives provides an advantage and significantly increases the antioxidant efficiency of HCAs in lactobacilli.

Similarly to the decarboxylation process, the expression of the *hcrAB* genes and hence the reduction process, is controlled by HcrR, a Lys-R type transcriptional regulator. The *hcrR* gene lies downstream of *hcrB* and is cotranscribed with *hcrA* and *hrcB* (Figure 2). The HcrR regulator participates and plays a key role in the reduction process, as shown by the absence of hydroxycinnamate reductase activity in a *L. plantarum* WCFS1 *hcrR* knockout mutant [37].

### 4.1. Variability in the HCA Reduction and Decarboxylation Metabolisms across Lactobacillus spp.

As mentioned above, it has been recently reported that the genetic complement for the metabolism of HCAs seems to be rather variable in *Lactobacillus* spp. [17]. Thus, while some of these bacteria apparently possess the complete gene toolkit required to metabolize HCAs via decarboxylation (*pad*, *vprA*) and reduction (*hcr*) pathways, others encode PAD but not HCR enzymes, while others lack PAD but encode HCR and a few hold PAD and VprA while others encode VprA but not PAD [17]. However, regarding the HCR enzymes, it must be reiterated that not all proposed putative enzymes have been biochemically validated to support their involvement in HCA reduction.

This comparative genomic analysis has identified homologs with different degrees of identity to *hrcA* or *hrcB* in heterofermentative lactobacilli. Some of the found *hrcB*-like genes have been proposed as novel putative phenolic acid reductases on the basis that they were variably overexpressed in the presence of different hydroxycinnamic acids (sinapic, ferulic or caffeic acids) [17]. One of these *hrcB*-like genes, named *par1*, was found in *F. rossiae* strains that lack HrcB and displayed the lowest degree of identity to *L. plantarum* WCFS 1 HcrB among the proposed putative HCR reductases. Interestingly, the deletion of *par1* rendered *F. rossiae* mutants unable to reduce the mentioned acids to their corresponding phenylpropionic acids, showing its involvement in the reduction process in this strain [17].

However, a transcriptomic study showed that *par1* homologs from *L. plantarum* WCFS1 (*lp_0952*, *lp_0055*) are not responsive to *p*-CA (in contrast to *hrcB*) [8]. In addition, *par1* homologs from other two *L. plantarum* strains (TMW 1.460 and FUA3584) were also not responsive to other HCAs (sinapic, ferulic or caffeic acids) [17]. These observations suggest that the sole presence of some *hrc* or *hcr*-like genes do not necessarily match with the reduction phenotype. It must also be noted that even the overexpression of these genes is not necessarily related to the reduction phenotype, as was shown in the case of cinnamic acid, a phenolic compound able to induce the expression of the *L. plantarum* HcrB, but not to act as a substrate for this enzyme [37]. Therefore, regarding HCR reductases, the genotype (presence or absence of the coding gene) and overexpression may not precisely forecast the phenotype. In this line, mutant approaches and biochemical characterization for other previously proposed putative reductases [17] are required in order to validate their contribution to the reduction process. In addition, it would be necessary to continue the biochemical characterization of confirmed HCR reductases, such as Par1, to further confirm their implication in the reduction process and bring new information on the scarcely studied oxidoreductases that act on carbon–carbon double bonds of HCAs.

In view of the variability of HCA metabolism across lactobacilli, the observed differences in the genetic toolkits for HCA metabolism among species and the high variability in the expression profile of these metabolic genes, new studies will probably be necessary to validate and ascribe a role to the proposed putative enzymes in the metabolism of HCAs.

### 4.2. HrcAB Reductase: Open Questions

HcrAB has been demonstrated to reduce HCAs; however, whole-genome transcription profiling revealed that *hcrAB* genes were significantly upregulated in the presence of the phenolic compounds oleuropein (OLE) [43] or gallic acid (GA) [44]. This induction is significant, not least since the *hrcAB* genes showed the highest induction among all the reductases whose expression were modulated by OLE. In the case of GA, HcrAB was the sole reductase induced in the presence of this hydroxybenzoic acid. Due to the structural differences between hydroxycinnamic acids, OLE and GA, the observed expression profiles provide hints that the activity of HcrAB phenolic reductase could be not restricted to hydroxycinnamic acids. Of note, it has been also observed that cinnammic acid also transcriptionally induces *hcrB* [37], albeit this acid does not apparently act as a substrate under the conditions assayed.

In addition, it is important to note that the current data on the transformation of HCAs into substituted phenyl-propionic acids by the *L. plantarum* HcrB have been obtained from *E. coli* extracts overexpressing this protein, as the pure HcrB protein, as mentioned above, underwent partial hydrolysis and substantially lost activity in the presence of oxygen [37]. This low activity is in agreement with previous observations reporting that the reductase activity on HCAs is about 100 times lower than the decarboxylase activity [19]. Altogether these results suggest that HcrB could be oxygen-sensitive, resembling strict oxygen-sensitive enoate reductases from *Clostridium acetobutylicum* that are able to aerobically reduce cinnamic acid and *p*-CA into 3-PPA and HPPA, respectively, in *E. coli* [45]. It has been proposed that the oxygen toxicity in the intracellular environment of *E. coli* is low due to the expression of cellular respiratory chain on the membrane or enzymes such as SOD [46,47], which prevents the inactivation of the oxygen sensitive enzymes. Extracts from *E. coli* that overexpress the *L. plantarum* HcrB contain SOD, an enzyme able to prevent the oxygen toxicity of superoxide radicals which, however, probably cannot be relieved in the pure HcrB enzyme preparation.

Furthermore, it should not be forgotten that reductions mediated by HcrB, which display NAD(P)H and flavin-binding domains, have been shown to require NADH and FMN as cofactors [37], which implies intramolecular electron transfer from NADH to the enzyme bound flavin FMN cofactor on the way to the final acceptor(s), the phenolic compound(s). The presence of oxygen, which is the best terminal electron acceptor in nature due to its high electronegativity (standard reduction potential of +1229 mV), can interfere with the electron transfer in the HcrB-catalyzed reaction and increase the rate of oxidation of HCAs, especially in the presence of traces of catalytic ubiquitous redox metals. In addition, the presence of oxygen can boost the competition for the cofactors required for the HcrB (NADH, FMN) by very competitive enzymes for NADH, particularly the NADH-oxidase [48,49], which could also explain the low prevalence of the reductase activity with respect to the decarboxylation in *L. plantarum*, at least under aerobic conditions.

Considering that in *L. plantarum*, (i) the partial HcrB hydrolysis, (ii) the low HcrB activity, (iii) the potential competition of other enzymes for the cofactors NADH and FMN and (iv) the much lower HCR activity compared to decarboxylase activity are all fostered under aerobic conditions, a physiological advantage of using HcrB in the presence of oxygen appears unlikely. Therefore, assays and characterization of HrcB and other putative reductases from lactobacilli under anaerobic conditions could provide valuable new information on the mechanism of action and the range of substrates that these enzymes can reduce.

In fact, the physiological importance of the phenolic acid reductase activity has been investigated in heterofermentative lactic acid bacteria [50,51] under anaerobic conditions in order to exclude other final acceptors, such as the oxygen which is plentiful in the environment or fructose which is a major carbohydrate for some of these bacteria. Under these conditions, phenolic acids have been shown to be used as external electron acceptors. The physiological advantages of this strategy include an increase in the oxidation of NADH (increase in the NAD+/NADH ratio) accompanied by an increase in acetate production with the concomitant accumulation of additional ATP.

## 5. Concluding Remarks

*Lactobacillus* spp. are able to transform hydroxycinnamic acids by decarboxylation and/or by reduction into different derivatives. Most of the genetically and biochemically supported knowledge on the enzymes involved in the metabolic conversions in lactobacilli arise from studies in *L. plantarum*. The decarboxylation of HCAs by lactobacilli to the corresponding vinyl derivatives is catalyzed by the inducible phenolic acid decarboxylase (PAD), which is controlled by the PadR Lys-R type regulator. These vinyl derivatives display lower acidity than their HCA precursors, are less effective in complexing transition metals and are less likely to increase their lipophilicity and to accelerate lipid oxidation than HCAs. The vinyl derivatives of HCAs can be further reduced by the vinylphenol reductase enzyme (VprA) into ethyl derivatives. This metabolic transformation is controlled by the Lys-R type regulator VprR and is rather limited among lactobacilli, as VprA is not widely distributed among these microorganisms and other lactic acid bacteria. Some lactobacilli seem to hold VprA but not PAD, and ethylphenol is produced more efficiently in *L. plantarum*, which holds *vprA* and *pad* genes, if vinylphenol is exogenously provided, with the decarboxylation by the PAD being the limiting factor for the production of ethylphenol. These data show that PAD and VprA can function independently and suggest that they are not necessarily steps of the same pathway. In addition to decarboxylation, some *Lactobacillus* spp. display the capacity to reduce hydroxycinnamic acids into phenylpropionic acids. To date, only one enzyme that catalyzes the reduction of HCAs, HcrB, has been genetically and biochemically characterized. Other putative phenolic reductases have recently been proposed to reduce HCAs, but only the product of *par1* has been demonstrated to be involved in HCA reduction in heterofermentative lactobacilli, albeit the encoded reductase has not been yet characterized. Despite the fact that decarboxylation provides a quick response on a huge scale to metabolize HCAs to relieve HCA stress to lactobacilli, some heterofermentative species of *Lactobacillus* only reduce HCAs, and some HCAs, such as *m*-coumaric acid, are only metabolized by reduction. The competitive metabolic advantage that these heterofermentative lactobacilli obtain under anaerobic conditions by using HCAs as external electron acceptors to regenerate NADH in the reduction reaction, and to gain additional ATP, can explain the preference of the reduction over the decarboxylation pathway to metabolize these phenolic compounds.

In conclusion, this review summarizes and critically discusses the updated knowledge on the molecular bases of hydroxycinnamic acid metabolism via decarboxylation and reduction pathways in lactobacilli. Whilst some crucial genes and enzymes involved in these metabolic conversions have been identified and characterized in detail, including their regulation and physiological significance, it has been proposed that the gene toolkit required to metabolize these phenolic compounds is rather variable across lactobacilli. This review sheds light on the missing puzzle pieces, in particular for the reduction pathway. Expansion of this field is essential to solidly identify and characterize novel hydroxycinnamic acid decarboxylating or reducing enzymes from lactobacilli. This will offer opportunities to provide molecular science-based innovations to produce and control the valuable compounds that are derived from these metabolic conversions. 

## Figures and Tables

**Table 1:** Structures and redox potentials of hydroxycinnamic acids.

	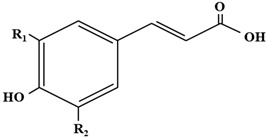		
**Compound**	**R_1_**	**R_2_**	***Ep* (mV)**
p-Coumaric acid	OH	H	+736
Caffeic acid	OH	H	+183
Ferulic acid	OCH_3_	H	+335
Sinapic acid	OCH_3_	OCH3	+188
